# Supplementary material for: P-TEFb promotes cell survival upon p53 activation by suppressing intrinsic apoptosis pathway
Source: Nucleic Acids Res. 2023 Feb 2;51(4):1687–706. doi: 10.1093/nar/gkad001 (PMC9976905; doi:10.1093/nar/gkad001)
Supplement: gkad001_Supplemental_File [file gkad001_supplemental_file.pdf]

Supplementary Data

P-TEFb promotes cell survival upon p53 activation by suppressing intrinsic apoptosis pathway

Zhijia Wang,[1] Monika Mačáková,[1,6] Andrii Bugai,[1,2,6] Sergey G. Kuznetsov,[3] Antti Hassinen,[4] Tina Lenasi,[1] Swapnil Potdar,[3] Caroline C. Friedel,[5] Matjaž Barborič [1,*]

[1]Department of Biochemistry and Developmental Biology, University of Helsinki, Helsinki FIN-00014, Finland

[2]Department of Molecular Biology and Genetics, Aarhus University, 8000 Aarhus C, Denmark

[3]High-Throughput Biomedicine Unit (HTB), Institute for Molecular Medicine Finland (FIMM), University of Helsinki, Helsinki FIN-00014, Finland

[4]High Content Imaging and Analysis Unit (HCA), Institute for Molecular Medicine Finland (FIMM), University of Helsinki, Helsinki FIN-00014, Finland

[5]Institute for Informatics, Ludwig-Maximilians-Universität München, 80333 Munich, Germany

[6]These authors contributed equally

[*]Correspondence: matjaz.barboric@helsinki.fi

Contents:

1. Supplementary Figures S1-S7

2. Supplementary Tables S1-S3

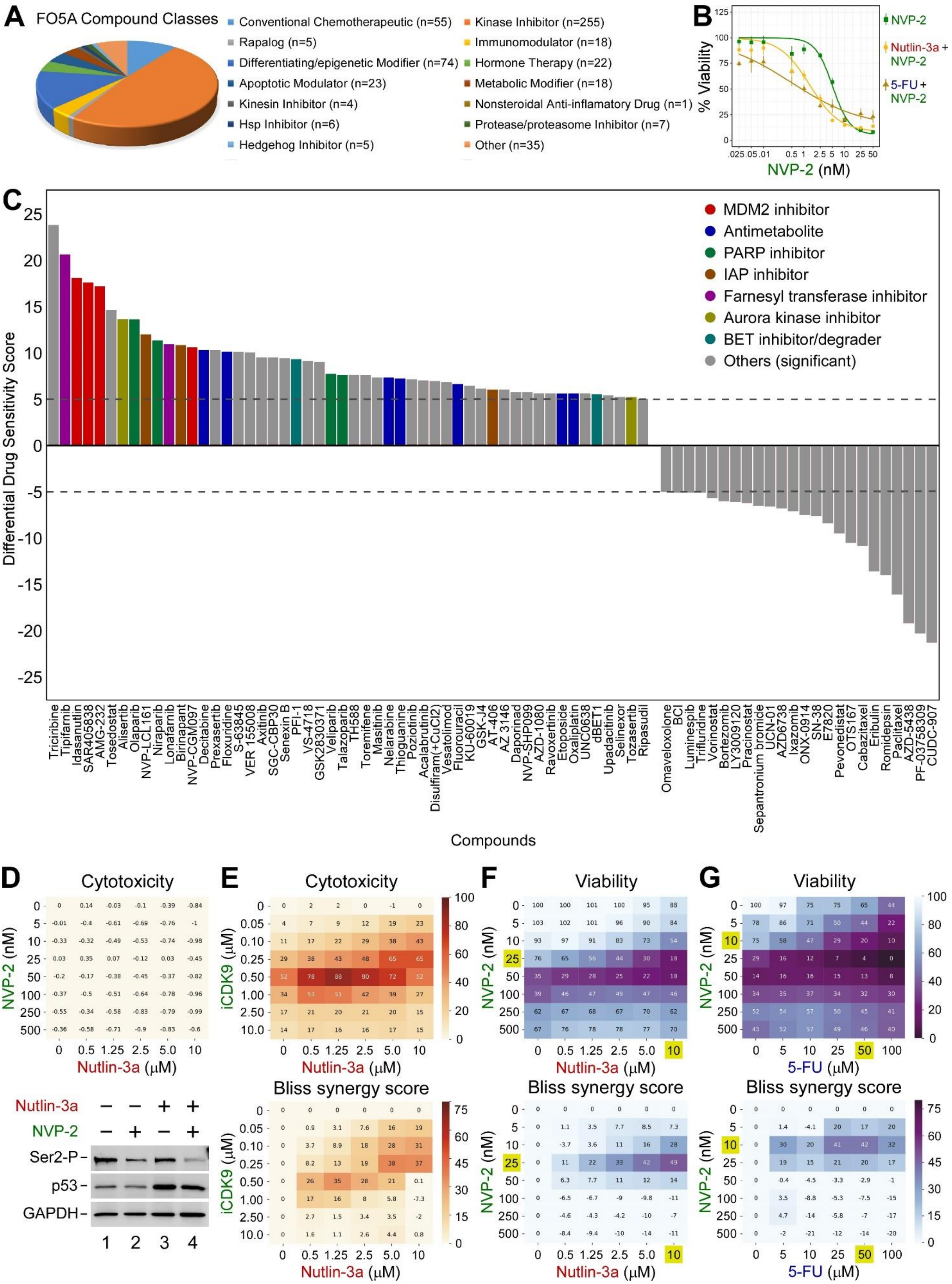

A
FO5A Compound Classes
Conventional Chemotherapeutic (n=55)
Kinase Inhibitor (n=255)
Rapalog (n=5)
Immunomodulator (n=18)
Differentiating/epigenetic Modifier (n=74)
Hormone Therapy (n=22)
Apoptotic Modulator (n=23)
Metabolic Modifier (n=18)
Kinesin Inhibitor (n=4)
Nonsteroidal Anti-inflamatory Drug (n=1)
Hsp Inhibitor (n=6)
Protease/proteasome Inhibitor (n=7)
Hedgehog Inhibitor (n=5)
Other (n=35)
B
% Viability
NVP-2 (nM)
NVP-2
Nutlin-3a + NVP-2
5-FU + NVP-2
C
Differential Drug Sensitivity Score
Compounds
MDM2 inhibitor
Antimetabolite
PARP inhibitor
IAP inhibitor
Farnesyl transferase inhibitor
Aurora kinase inhibitor
BET inhibitor/degrader
Others (significant)
Triciribine
Tipifarnib
Idasanutlin
SAR405838
AMG-232
Tosedostat
Alisertib
Olaparib
NVP-LCL161
Niraparib
Lonafarnib
Birinapant
NVP-CGM097
Decitabine
Prexasertib
Floxuridine
S-63845
VER 155008
Axitinib
SGC-CBP30
Senexin B
PFI-1
VS-4718
GSK2830371
Veliparib
Talazoparib
TH588
Toremifene
Masitinib
Nelarabine
Thioguanine
Poziotinib
Acalabrutinib
Disulfiram(+CuCl2)
Vesatolimod
Fluorouracil
KU-60019
GSK-J4
AT-406
AZ 3146
Daporinad
NVP-SHP099
AZD-1080
Ravoxertinib
Etoposide
Oxaliplatin
UNC0638
dBET1
Upadacitinib
Selinexor
Tozasertib
Ripasudil
Omaveloxolone
BCI
Luminespib
Trifluridine
Vorinostat
Bortezomib
LY3009120
Pracinostat
Sepantronium bromide
UCN-01
AZD6738
Ixazomib
ONX-0914
SN-38
E7820
Pevonedistat
OTS167
Cabazitaxel
Eribulin
Romidepsin
Paclitaxel
AZD-5438
PF-03758309
CUDC-907
D
Cytotoxicity
NVP-2 (nM)
Nutlin-3a (μM)
Nutlin-3a
NVP-2
Ser2-P
p53
GAPDH
1 2 3 4
E
Cytotoxicity
iCDK9 (μM)
Nutlin-3a (μM)
Bliss synergy score
F
Viability
NVP-2 (nM)
Nutlin-3a (μM)
Bliss synergy score
G
Viability
NVP-2 (nM)
5-FU (μM)
Bliss synergy score

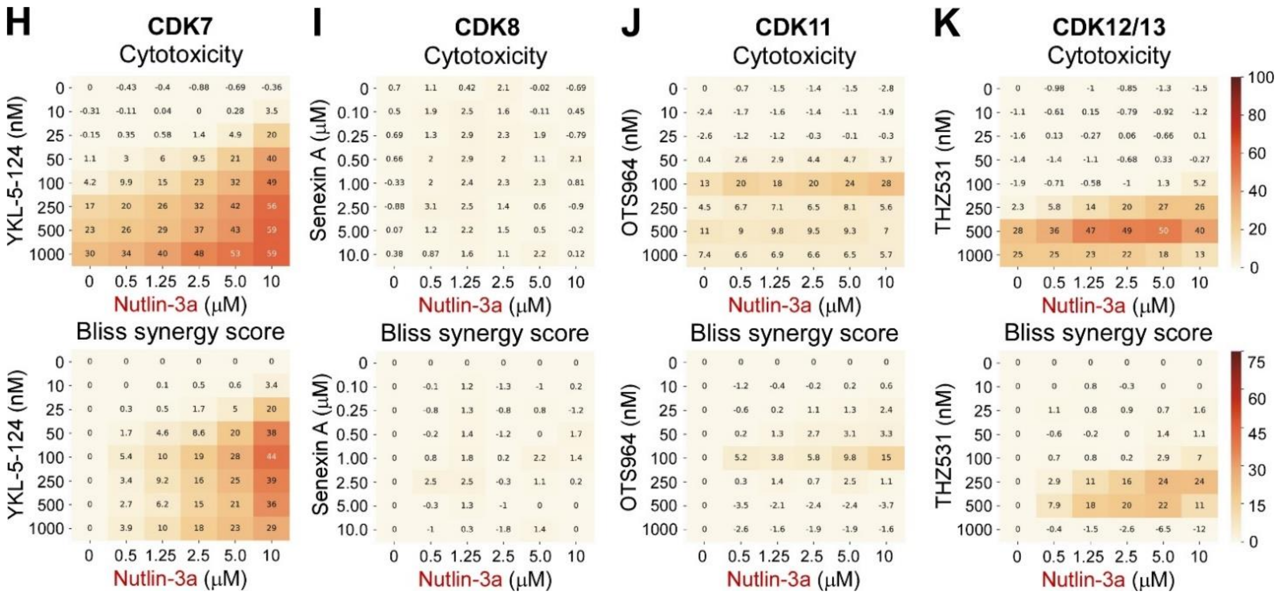

Supplementary Figure S1. Selective inhibition of CDK7 and P-TEFb but not other tCDKs is synthetic-lethal with MDM2 inhibitor Nutlin-3a.

(A) Compound classes of the FO5A oncology library. Pie chart represents classes of investigational and clinical anti-cancer compounds listed on the right.

(B) Dose-response viability curves of HCT116 cells treated with increasing doses of NVP-2 (green) alone and in combination with Nutlin-3a (10 μM; light gold) or 5-Fluorouracil (5-FU; 25 μM, dark gold) as indicated. Viability values obtained at 72 hr of the treatments using CellTiter-Glo 2.0 Cell Viability Assay were normalized to the values of DMSO-treated cells and are presented as percentages of the maximum viability which was set at 100 %. Results are presented as the average ± s.d. (n = 2).

(C) Waterfall plot representing results of the screen. Compounds with dDSS values ≥ 5 and ≤ -5 were considered as significant and depicted according to the legend on the right.

(D) (Top) 8 × 6 cytotoxicity matrix with combinatorial titrations of NVP-2 (green) with Nutlin-3a (red) at indicated doses in non-transformed CCD 841 CoN colon epithelial cells. Cytotoxicity values obtained at 48 hr of the treatments using CellTox Green Cytotoxicity Assay were normalized to the values of DMSO-treated cells and are presented as percentages of the maximum cytotoxicity which was set at 100 %. Results represent the average of independent experiments (n = 3). (Bottom) CCD 841 CoN cells were treated with the indicated combinations of NVP-2 (10 nM) and Nutlin-3a (10 μM) for 8 hr prior to preparation of whole cell extracts and detection of the indicated proteins by Western blotting.

(E, H-K) 8 × 6 matrices with combinatorial titrations of iCDK9 (green) and the indicated tCDK inhibitors (black) with Nutlin-3a (red) at indicated doses to test for the synthetic lethality of compounds in HCT116 cells, depicting cytotoxicity (top) and synergy (bottom) of the combinations. Targeted tCDKs are indicated on top. Cytotoxicity values obtained at 48 hr of the treatments using CellTox Green Cytotoxicity Assay were normalized to the values of DMSO-treated cells and are presented as percentages of the maximum cytotoxicity which was set at 100 %. Results represent the average of independent experiments (n = 3).

(F,G) 8 × 6 matrices with combinatorial titrations of NVP-2 (green) with Nutlin-3a (red) and 5-Fluorouracil (5-FU; blue) at indicated doses to test for the synthetic lethality of compounds in HCT116 cells, depicting viability (top) and synergy (bottom) of the combinations. Viability values obtained at 48 hr of the treatments using CellTiter-Glo 2.0 Cell Viability Assay were normalized to the values of DMSO-treated cells and are presented as percentages of the maximum viability which was set at 100 %. Results represent the average of independent experiments (n = 3). Combinations with the highest Bliss synergy scores in HCT116 cells are highlighted (gold).

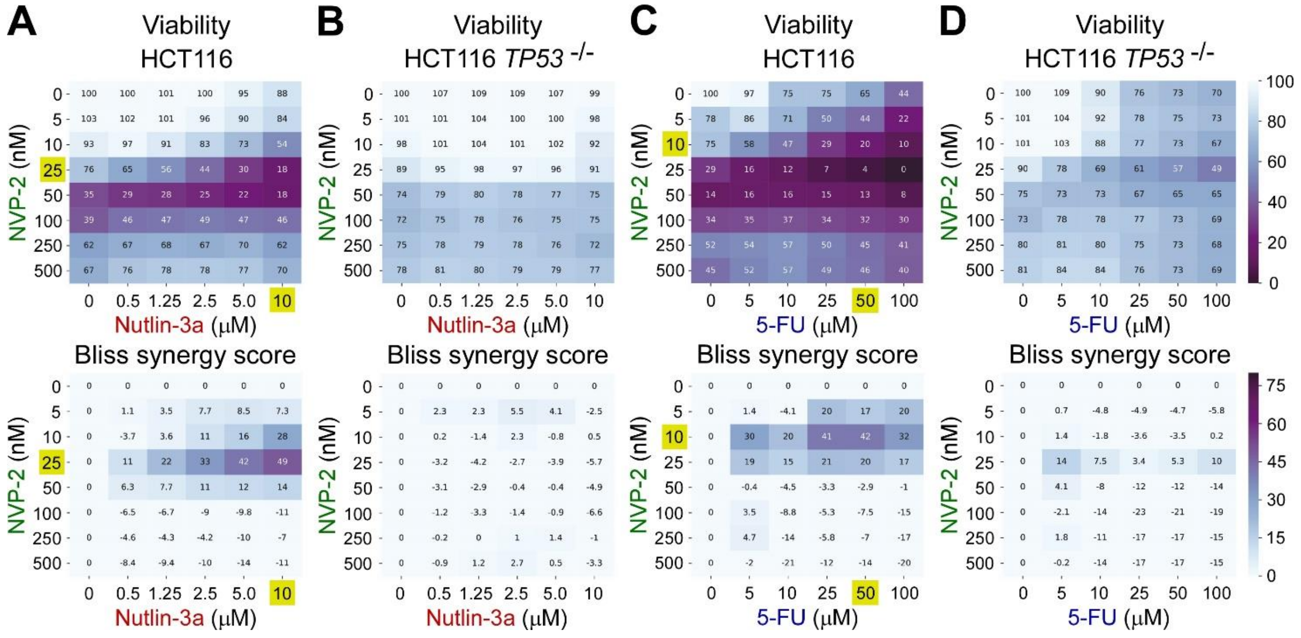

Supplementary Figure S2. Synthetic lethality of Nutlin-3a and antimetabolites with P-TEFb inhibitor NVP-2 is p53-dependent.

(A-D) 8 × 6 matrices with combinatorial titrations of NVP-2 (green) with Nutlin-3a (red) and 5-Fluorouracil (5-FU; blue) at indicated doses to test for the synthetic lethality of compounds in HCT116 and HCT116 *TP53* $^{-/-}$ cells, depicting viability (top) and synergy (bottom) of the combinations. Viability values obtained at 48 hr of the treatments using CellTiter-Glo 2.0 Cell Viability Assay were normalized to the values of DMSO-treated cells and are presented as percentages of the maximum viability which was set at 100 %. Results represent the average of independent experiments (n = 3). Combinations with the highest Bliss synergy scores in HCT116 cells are highlighted (gold).

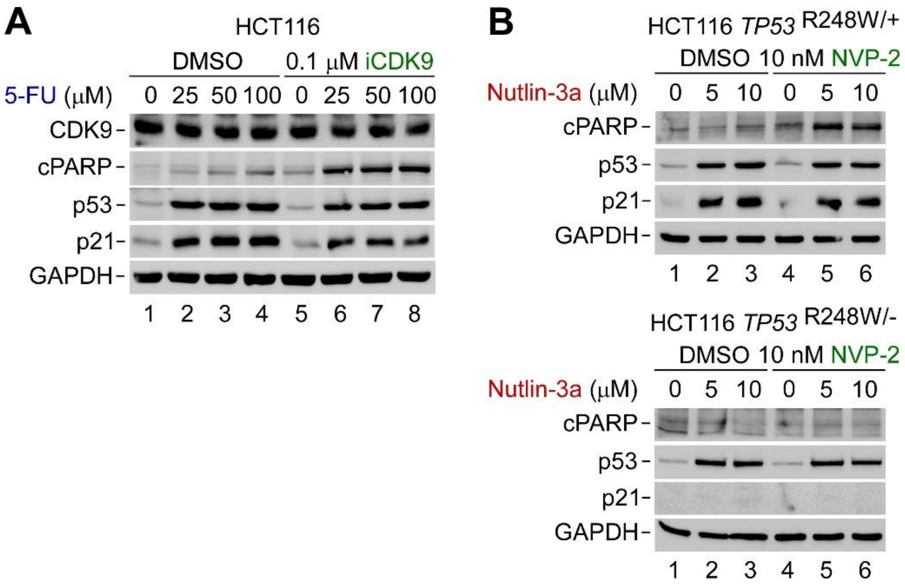

Supplementary Figure S3. Activation of apoptosis underlies p53-dependent synthetic lethality of p53 activation and P-TEFb inhibition.

(A,B) HCT116, HCT116 *TP53* $^{R248W/+}$ and HCT116 *TP53* $^{R248W/-}$ cells were treated with DMSO and indicated combinations and doses of CDK9 inhibitors (green), Nutlin-3a (red), and 5-Fluorouracil (5-FU; blue) for 24 hr prior to preparation of whole cell extracts and detection of the indicated proteins by Western blotting.

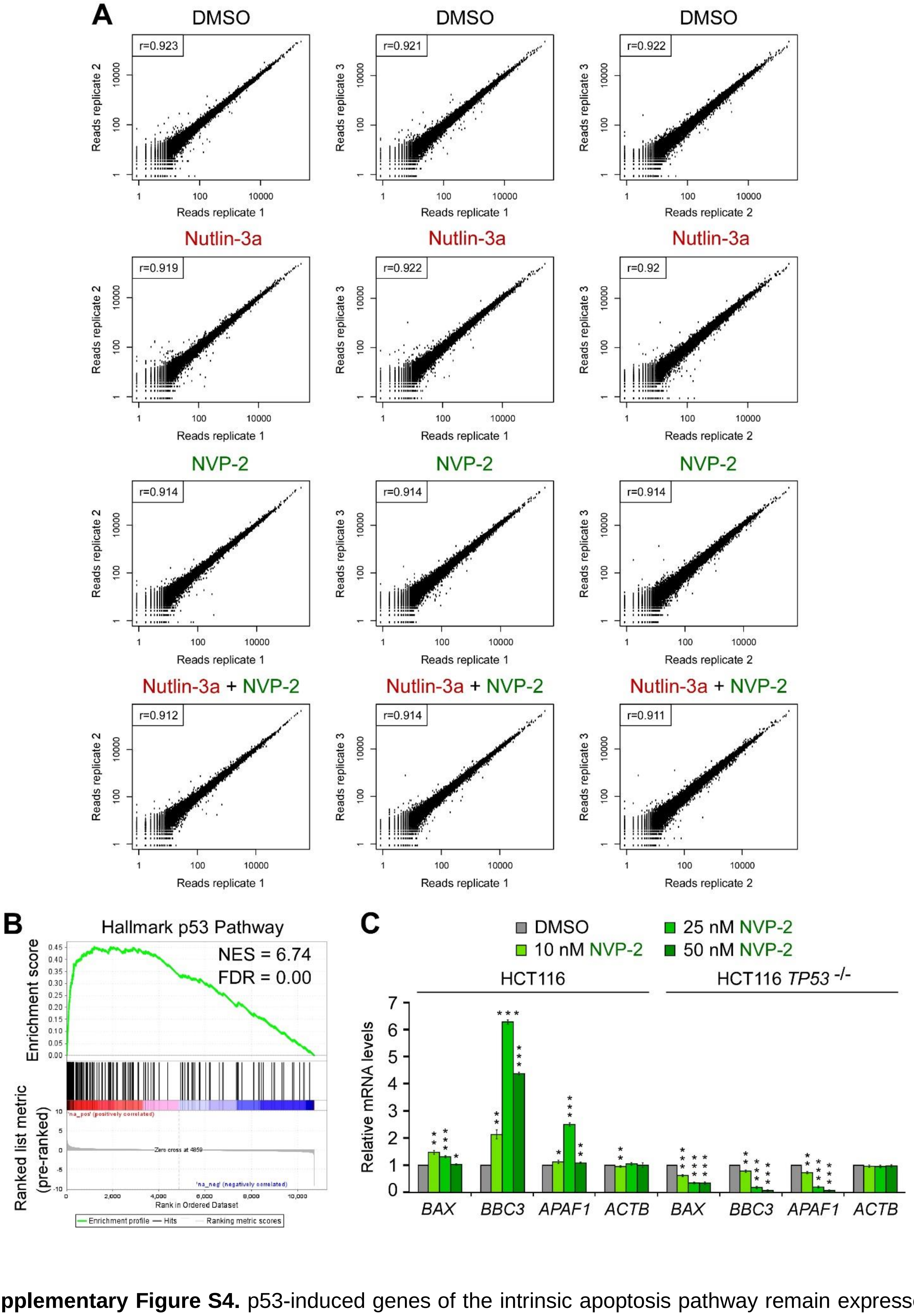

Supplementary Figure S4. p53-induced genes of the intrinsic apoptosis pathway remain expressed under sub-lethal inhibition of P-TEFb.

(A) Scatterplots comparing raw read counts of all annotated genes from RNA-seq data sets (n = 3) are shown and spearman correlation (r) is indicated. HCT116 cells were treated for 8 hr as indicated on top of each graph.

(B) Gene set enrichment analysis of the protein-coding gene data set regulated by Nutlin-3a. The top gene set is shown. NES, normalized enrichment score. FDR, false discovery rate.

(C) HCT116 and HCT116 *TP53* $^{-/-}$ cells were treated with DMSO (grey) and increasing doses of NVP-2 (green) for 24 hr prior to quantifying mRNA levels of the indicated genes with RT-qPCR. Results normalized to the levels of *GAPDH* mRNA and DMSO-treated cells are presented as the mean ± s.e.m. (n = 3). *, P < 0.05; **, P < 0.01; ***, P < 0.001, determined by Student's *t* test.

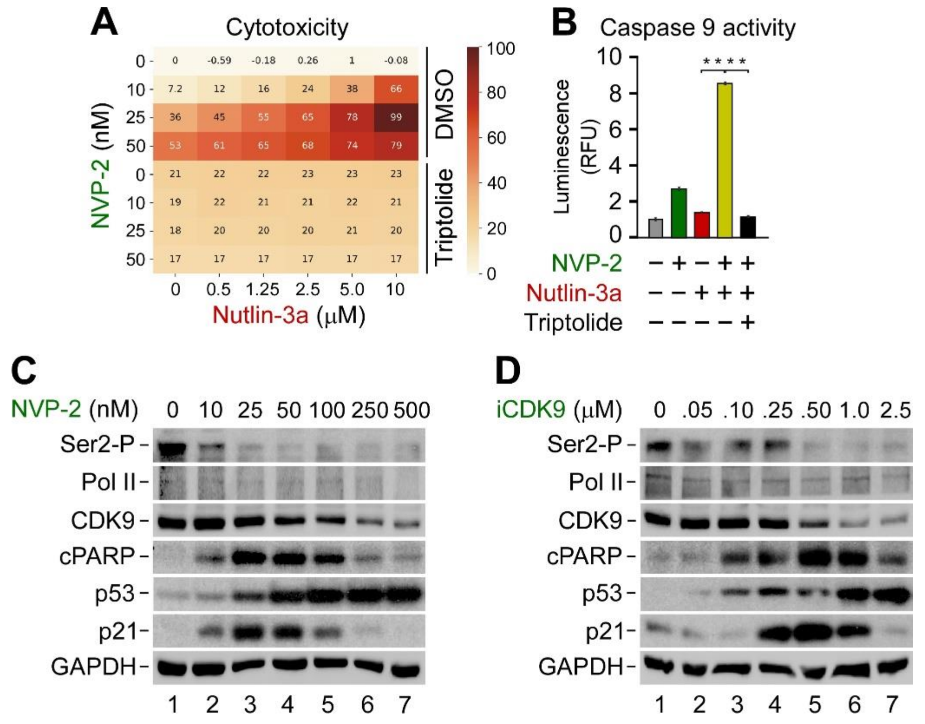

Supplementary Figure S5. Synthetic lethality of non-genotoxic p53 activation and P-TEFb inhibition depends on intrinsic apoptosis pathway and on-going Pol II transcription.

(A) 4 × 6 cytotoxicity matrices with combinatorial titrations of Nutlin-3a (red) with NVP-2 (green) at indicated doses of HCT116 cells co-treated with DMSO or Triptolide (1 μM) as indicated. Cytotoxicity values obtained at 48 hr of the treatments using CellTox Green Cytotoxicity Assay were normalized to the values of DMSO-treated cells and are presented as percentages of the maximum cytotoxicity which was set at 100 %.

(B) Activity of Caspase 9 measured using Caspase-Glo 9 Assay in whole cell extracts of HCT116 cells treated with DMSO (grey), NVP-2 (3 nM; green) and Nutlin-3a (10 μM; red) alone and in combination (gold), and with Triptolide (1 μM; black) as indicated for 18 hr. Results are presented as luminescence values relative to the values of DMSO-treated cells and plotted as the mean ± s.e.m. (n = 4). **, $P < 0.01$, determined by Student's *t* test.

(C,D) HCT116 cells were treated with DMSO and increasing doses of NVP-2 and iCDK9 (green) for 24 hr prior to preparation of whole cell extracts and detection of the indicated proteins by Western blotting.

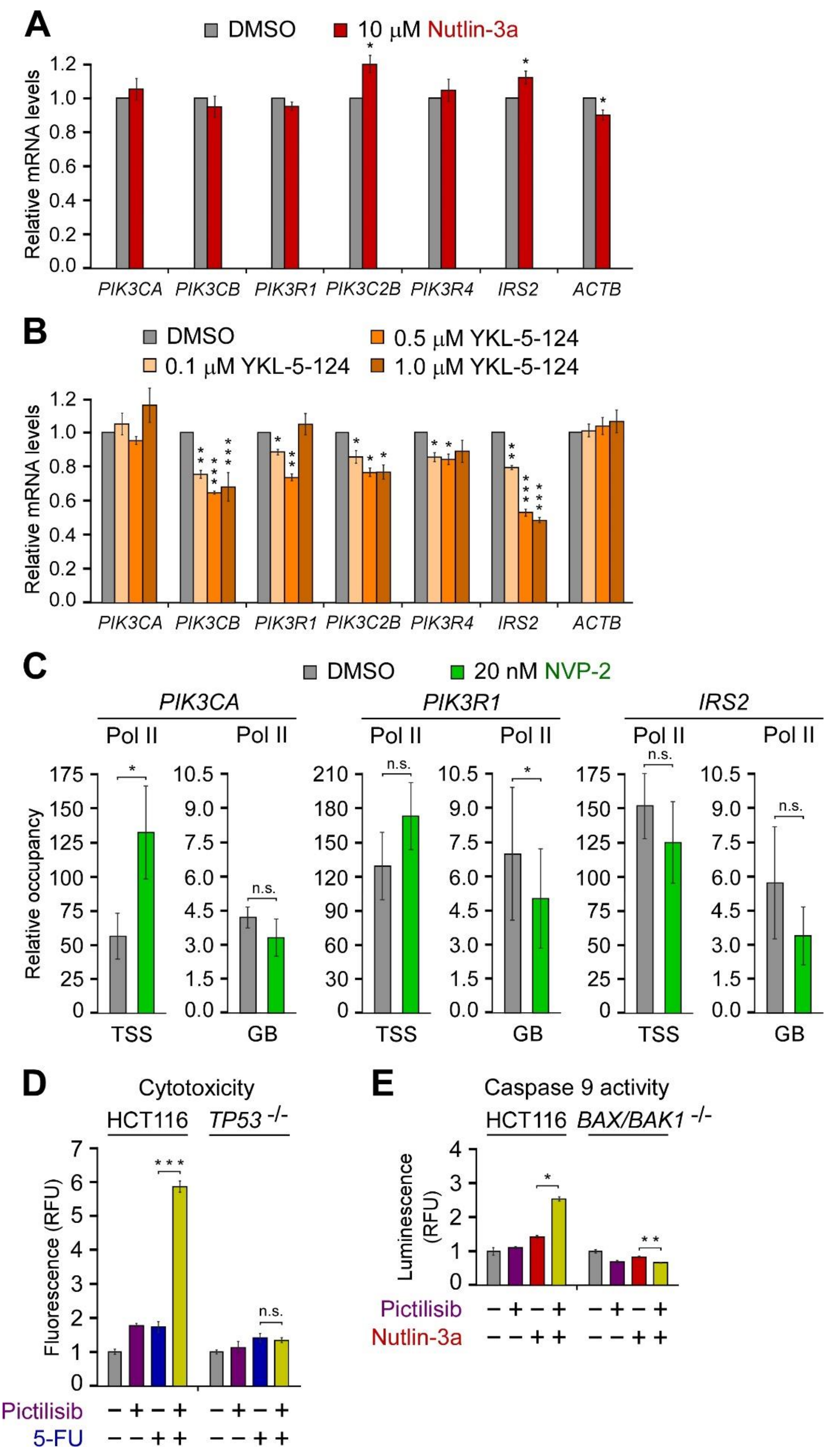

Supplementary Figure S6. Repression of genes encoding key components of the pro-survival PI3K-AKT pathway is a driver of the synthetic lethality of p53 activation and P-TEFb inhibition.

(A) HCT116 cells were treated with DMSO (grey) and Nutlin-3a (10 μM; red) for 24 hr prior to quantifying mRNA levels of the indicated genes with RT-qPCR. Results normalized to the levels of *GAPDH* mRNA and DMSO-treated cells are presented as the mean ± s.e.m. (n = 3). *, P < 0.05, determined by Student's *t* test.

(B) HCT116 cells were treated with DMSO (grey) and increasing doses of YKL-5-124 (orange) for 8 hr prior to quantifying mRNA levels of the indicated genes with RT-qPCR. Results normalized to the levels of *GAPDH* mRNA and DMSO-treated cells are presented as the mean ± s.e.m. (n = 3). *, P < 0.05; **, P < 0.01; ***, P < 0.001, determined by Student's *t* test.

(C) HCT116 cells were treated with DMSO (grey) and NVP-2 (20 nM; green) for 3 hr prior to determining the levels of total Pol II at transcription start site (TSS) and gene body (GB) of the indicated genes with ChIP-qPCR. Results normalized to the values of IgG in DMSO-treated cells are presented as the mean ± s.e.m. (n = 2). *, P < 0.05; n.s., non-significant, determined by Student's *t* test.

(D) Cytotoxicity of HCT116 and HCT116 *TP53* $^{-/-}$ cells treated with DMSO (grey), Pictilisib (1 μM; magenta), and 5-Fluorouracil (5-FU; 25 μM) (blue) alone and in combination (gold) as indicated for 48 hr measured using CellTox Green Cytotoxicity Assay. Results are presented as fluorescence values relative to the values of DMSO-treated cells and plotted as the mean ± s.e.m. (n = 3). ***, P < 0.001; n.s., non-significant, determined by Student's *t* test.

(E) Activity of Caspase 9 measured using Caspase-Glo 9 Assay in whole cell extracts of HCT116 and HCT116 *BAX*/*BAK1* $^{-/-}$ cells treated with DMSO (grey), Pictilisib (1 μM; magenta), and Nutlin-3a (10 μM; red) alone and in combination (gold) as indicated for 18 hr. Results are presented as luminescence values relative to the values of DMSO-treated cells and plotted as the mean ± s.e.m. (n = 3). *, P < 0.05; **, P < 0.01; n.s., non-significant, determined by Student's *t* test.

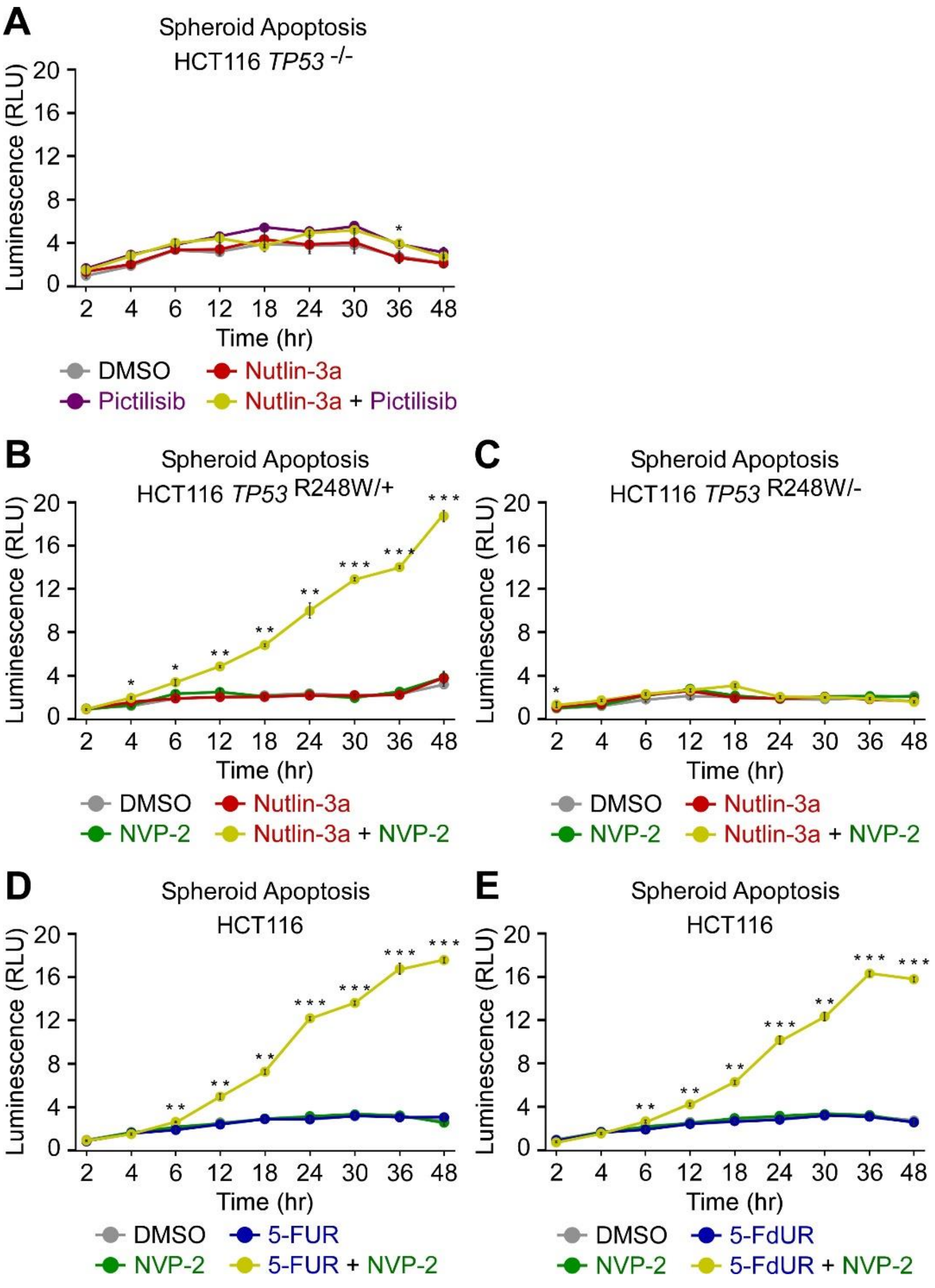

Supplementary Figure S7. Combination treatments within the framework of p53 activation and P-TEFb inhibition trigger apoptosis of HCT116 spheroid cultures.

(A-E) Apoptosis of HCT116 *TP53* $^{-/-}$, HCT116 *TP53* $^{R248W/+}$, HCT116 *TP53* $^{R248W/-}$ and HCT116 cell spheroid cultures treated with DMSO (grey), Nutlin-3a (10 μM; red), Pictilisib (0.5 μM; magenta), NVP-2 (10 nM; green), 5-fluorouridine (5-FUR, 0.5 μM; blue) and 5-fluorodeoxyuridine (5-FdUR, 0.15 μM; blue) alone and in combinations (gold) as indicated. Spheroids were formed for 48 hr prior to the treatments. Results obtained at the time points indicated below the graphs using RealTime-Glo Annexin V Apoptosis and Necrosis Assay are presented as luminescence values relative to the levels of DMSO-treated cells at 2 hr and plotted as the mean ± s.e.m. (n = 3). *, $P < 0.05$; **, $P < 0.01$; ***, $P < 0.001$, determined by Student's *t* test using Nutlin-3a and Nutlin-3a + Pictilisib (A), Nutlin-3a and Nutlin-3a + NVP-2 (B, C), 5-FUR and 5-FUR + NVP-2 (D), 5-FdUR and 5-FdUR + NVP-2 (E) data sets.

Antibody	Source	Identifier
Mouse monoclonal anti-CDK9	Santa Cruz Biotechnology	Cat#sc-13130; RRID: AB_627245
Mouse monoclonal anti-p53	Santa Cruz Biotechnology	Cat#sc-126; RRID: AB_628082
Rabbit polyclonal anti-Cleaved PARP	Cell Signaling Technology	Cat#9541; RRID: AB_331426
Mouse monoclonal anti-p21	Santa Cruz Biotechnology	Cat#sc-53870; RRID: AB_785026
Mouse monoclonal anti-GAPDH	Santa Cruz Biotechnology	Cat#sc-32233; RRID: AB_627679
Rabbit polyclonal anti-RNA polymerase II CTD repeat YSPTSPS (phospho S2)	Abcam	Cat#ab5095; RRID: AB_304749
Rabbit polyclonal anti-RNA polymerase II CTD repeat YSPTSPS (phospho S5)	Abcam	Cat#ab5131; RRID: AB_449369
Rabbit monoclonal anti-RNA Polymerase II RPB1 NTD	Cell Signaling Technology	Cat#14958S; RRID: AB_2687876
Normal rabbit IgG	Santa Cruz Biotechnology	Cat#sc-2027; RRID: AB_737197
Mouse monoclonal anti-RNA Polymerase II RPB1 NTD	Santa Cruz Biotechnology	Cat#sc-17798; RRID: AB_677355
Mouse monoclonal anti-p110a	Santa Cruz Biotechnology	Cat#sc-293172; RRID: AB_2847954
Mouse monoclonal anti-p110β	Santa Cruz Biotechnology	Cat#sc-376641; RRID: AB_11150840
Mouse monoclonal anti-p85a	Santa Cruz Biotechnology	Cat#sc-1637; RRID: AB_628126
Anti-IRS-2	Santa Cruz Biotechnology	Cat#sc-390761
Mouse monoclonal anti-pan-Akt	Santa Cruz Biotechnology	Cat#sc-81434; RRID: AB_1118808
Rabbit monoclonal anti-phospho-Akt (Ser473)	Cell Signaling Technology	Cat#4060; RRID: AB_2315049
Rabbit monoclonal anti-phospho-p44/42 MAPK (Erk1/2) (Thr202/Tyr204)	Cell Signaling Technology	Cat#4376; RRID: AB_331772
Rabbit monoclonal anti-p44/42 MAPK (Erk1/2)	Cell Signaling Technology	Cat#4695; RRID: AB_390779

Supplementary Table S1A. Antibodies used in the study.

Chemical	Source	Identifier
FO5A oncology library	FIMM	N/A
NVP-2	MedChemExpress	Cat#HY-12214A
THAL-SNS-032	Nathanael S. Gray Laboratory (Stanford University)	N/A
i-CDK9	Qiang Zhou Laboratory (UC Berkeley)	N/A
Nutlin-3a	MedChemExpress	Cat#HY-10029
5-fluorouracil	Selleck Chemicals	Cat#S1209
5-fluorouridine	MedChemExpress	Cat#HY-107856
5-fluorodeoxyuridine	MedChemExpress	Cat#HY-B0097
YKL-5-124	Nathanael S. Gray Laboratory (Stanford University)	N/A
Senexin A	Selleck Chemicals	Cat#S8520
OTS964	Selleck Chemicals	Cat#S7648
THZ531	MedChemExpress	Cat#HY-103618
Triptolide	Selleck Chemicals	Cat#S3604
Pictilisib	MedChemExpress	Cat#HY-50094
DT-061	Jukka Westermarck Laboratory (University of Turku)	N/A
EDTA-free Protease Inhibitor Cocktail	Sigma	Cat#11873580001
Random hexamers	Thermo Fisher Scientific	Cat#N8080127
TRI Reagent	Sigma	Cat#T9424
Calcein AM	Invitrogen	Cat#L3224A
DRAQ7	Invitrogen	Cat#D15106
Hoechst 33342	Invitrogen	Cat#R37605

Supplementary Table S1B. Chemicals used in the study.

Assay	Source	Identifier
CellTox™ Green Cytotoxicity Assay	Promega	Cat#G8731
CellTiter-Glo® 2.0 Cell Viability Assay	Promega	Cat#G242
RealTime-Glo Annexin V Apoptosis and Necrosis Assay	Promega	Cat#JA1011
Caspase-Glo® 9 Assay	Promega	Cat#G8210
RNeasy Mini Kit (50)	Qiagen	Cat#74104
NEBNext Ultra Directional RNA Library Prep Kit	New England Biolabs	Cat#E7420
NEBNext Poly(A) mRNA Magnetic Isolation Module	New England Biolabs	Cat#E7490
M-MLV reverse transcriptase	Thermo Fisher Scientific	Cat#28025-013
Turbo DNA-freeTM kit	Thermo Fisher Scientific	Cat#AM1907
Dynabeads Protein G	Thermo Fisher Scientific	Cat#10004D
FastStart Universal SYBR Green QPCR Master (Rox)	Sigma	Cat#4913914001
Mycoplasmacheck detection kit	Eurofins	Cat#50400400

Supplementary Table S1C. Commercial assays used in the study.

Cell line	Source	Identifier
HCT116 and HCT116 *TP53* $^{-/-}$	Joaquin M. Espinosa Laboratory (University of Colorado)	N/A
HCT116 *TP53* $^{R248W/+}$ and HCT116 *TP53* $^{R248W/-}$	Bert Vogelstein Laboratory (John Hopkins University)	N/A
HCT116 *BAX*/*BAK1* $^{-/-}$	Ana J. Garcia-Saez Laboratory (University of Cologne)	N/A
CCD 841 CoN	ATCC	Cat#CRL-1790

Supplementary Table S1D. Cell lines used in the study.

Software	Source	Identifier
FastQC	Babraham Bioinformatics	http://www.bioinformatics.babraham.ac.uk/ projects/fastqc/)
ContextMap v2.7.9	(Bonfert et al., 2015)	https://www.bio.ifi.lmu.de/software/contextmap/index.html
featureCounts	(Liao et al., 2014)	http://bioinf.wehi.edu.au/featureCounts/
edgeR	(Robinson et al., 2010)	https://bioconductor.org/packages/release/bioc/html/ edgeR.html
Molecular Signatures Database v6.0	GSEA - Broad Institute (Subramanian et al., 2005)	http://software.broadinstitute.org/gsea/msigdb/index.jsp
Harmony v4.9 High-Content Imaging and Analysis Software	PerkinElmer	N/A
MxPro QPCR Software v4.10	Stratagene	N/A

Supplementary Table S1E. Software and algorithms used in the study.

Gene	Primer Sequence (5’-3’)	Primer Site
BAX	TTCTGACGGCAACTTCAACT	Exon 4
	CAGCCCATGATGGTTCTGAT	Exon 5
BBC3	CGACCTCAACGCACAGTA	Exon 2
	GGAGTCCCATGATGAGATTGTA	Exon 3
APAF1	TGGAGTCTCCCAGTCTTGT	Exon 1
	CCATCTTCCCTCAGATCTTTCTC	Exon 2
PIK3CA	GGGACCTCAATTCACCTCATAG	Exon 3
	TGGAGAAACTATTACCCAGATCAC	Exon 4
PIK3CB	CATGAACCATCCATCCCTGAA	Exon 5
	TAGGAGACACTTGAAAGCTAAACA	Exon 6
PIK3R1	CCAGCAACCTGGCAGAATTA	Exon 4
	GTAAGTCCAGGAGATAGCGTTTG	Exon 5
PIK3C2B	CAGAAGGATCCCAGTGTCTTG	Exon 9/10
	GAAGTCTGCGTTGAATGTGTTG	Exon 11
PIK3R4	GCCAGTTAGGACTGCTACAAA	Exon 7
	ATAAATCCCACGGCACCATAA	Exon 8
IRS2	GACTTCTTGTCCCACCACTT	Exon 1
	GACATGTGACATCCTGGTGATA	Exon 2
ACTB	ACTTAGTTGCGTTACACCCTTTCT	Exon 6
	GACTGCTGTCACCTTCACCGT	Exon 6
GAPDH	CGACCACTTTGTCAAGCTCA	Exon 8
	AGGGGAGATTCAGTGTGGTG	Exon 9

Supplementary Table S2. DNA oligonucleotides used in RT-qPCR assay.

Gene Site	Primer Sequence (5’-3’)
PIK3CA TSS	TCTCCTAGCTGCAGAGG
	GGAGTAGAAGCAAAGGAAGAG
PIK3CA GB	CAACAGCCACACACTACA
	CACCGACAGACTCATCTAAC
PIK3R1 TSS	GCTGGAGCGGAGTTGGA
	ATCCGTGCTTGCCGTCT
PIK3R1 GB	GAAGCGAGATGGCACTTT
	AGTTTGCTGGAGATACATACAC
IRS2 TSS	CTGTGTGTGCCTGCGTAA
	CCGCACAGTGAGTAACACAT
IRS2 GB	TCCTAGCTGTGGGTTAGAG
	TGTGACATCCTGGTGATAAAG

Supplementary Table S3. DNA oligonucleotides used in ChIP-qPCR assay.
